# Heads Up! Interlinked Amyloidogenic and Axonal Transport Pathways in Concussion-Induced Neurodegeneration

**DOI:** 10.1177/26331055221129641

**Published:** 2022-10-17

**Authors:** Angels Almenar-Queralt, Rodrigo dos Santos Chaves, Ester J. Kwon, Sameer B Shah

**Affiliations:** 1 Department of Pediatrics, University of California—San Diego, La Jolla, CA, USA; 2 Sanford Consortium for Regenerative Medicine, University of California—San Diego, La Jolla, CA, USA; 3 Department of Orthopaedic Surgery, University of California—San Diego, La Jolla, CA, USA; 4 Department of Bioengineering, University of California—San Diego, La Jolla, CA, USA; 5 Research Division, VA San Diego Healthcare System, San Diego, CA, USA

**Keywords:** Alzheimer’s, neurodegeneration, neurotrauma, amyloid precursor protein, amyloid beta

## Abstract

Mild traumatic brain injury (mTBI), a condition in which brain function is transiently disrupted by a mechanical force, is a major risk factor for developing Alzheimer’s disease (AD) and other neurodegenerative conditions. In this commentary, we summarize recent findings in human neurons derived from induced pluripotent stem cells, detailing early neuronal events following mild injury that may seed future neurodegeneration. In particular, we discuss interlinked relationships between mTBI and several biological pathways hypothesized to underlie AD progression, including amyloidogenic cleavage of amyloid precursor protein (APP), impairment of axonal transport, and the development of APP-associated axonal swellings. We also describe the implications of these findings for future mechanistic and translational studies.

**COMMENT ON:** Chaves RS, Tran M, Holder AR, Balcer AM, Dickey AM, Roberts EA, Bober BG, Gutierrez E, Head BP, Groisman A, Goldstein LSB, Almenar-Queralt A, Shah SB. Amyloidogenic Processing of Amyloid Precursor Protein Drives Stretch-Induced Disruption of Axonal Transport in hiPSC-Derived Neurons. J Neurosci. 2021 Dec 8;41(49):10034-10053. doi: 10.1523/JNEUROSCI.2553-20.2021. Epub 2021 Oct 18. PMID: 34663629; PMCID: PMC8660048.

## Background

Mild traumatic brain injury (mTBI), in which a mechanical force temporarily disrupts brain function (eg, a concussion), affects millions of people worldwide. Concussions result in transient neurological symptoms including headache, dizziness, feelings of confusion, and problems with concentration or memory. Although these symptoms typically resolve rapidly, individuals who have experienced mTBI are at increased risk for developing neurodegenerative disorders such as Alzheimer’s disease (AD) later in life, even after a single injury event.^1,2^ Yet, our ability to identify molecular links between mTBI and AD is limited, especially in humans. Major challenges include an inability to identify individuals who may be susceptible to mTBI-associated AD, poor prognostic capabilities due to ambiguities in assessing TBI severity, the impossibility of procuring live human brain tissue at different time points after injury, and a lack of in vitro human models to examine the spatiotemporal sequelae following injury. These knowledge gaps challenge the discovery and the screening of effective therapies to prevent mTBI-induced neurodegeneration.

While tau pathology, most notably tau phosphorylation, is viewed as a general hallmark of TBI, a key pathological feature that distinguishes TBI-induced AD from other neurodegenerative conditions is increased production of beta amyloid peptide (Aβ).^3^ Generation of Aβ results from the sequential cleavage of Amyloid Precursor Protein (APP) by β- and then γ-secretase, in a process known as amyloidogenic APP cleavage. In addition, axonal swellings filled with APP, suggestive of impaired axonal transport, are a well-documented morphological phenotype within injured regions of the brain and serve as a pathological marker for TBI-induced axonal damage.^4^ Previous studies suggest that altered amyloidogenic processing of APP and impaired APP axonal transport are interlinked, and both have been proposed as phenomena underlying generalized AD initiation and progression.^5^ However, whether and how these cellular processes are affected in human neurons following mTBI remains unexplored. A better understanding of potential changes in APP metabolism and/or APP axonal transport induced by mild injury may offer insights into how mTBI initiates the pathophysiological progression of AD-related outcomes. Additionally, lessons learned from TBI-associated AD-related outcomes could shed light into understanding the etiology of late onset AD, the most common form of dementia in the elderly. The lack of tools to longitudinally examine outcomes at sufficient spatial and temporal resolution in human neurons is a major barrier to interrogating such questions. This barrier is compounded by limitations of animal models (eg, the propensity for transgenic rodent models of AD to develop amyloid pathology independent of injury) and shortcomings of in vitro models (eg, the paucity of human model systems and the predominance of models simulating severe and lethal, as opposed to mild, neuronal trauma).

## APP Amyloidogenic Cleavage and Transport Impacted Early by Mild Neuronal Injury

**Figure 1.**
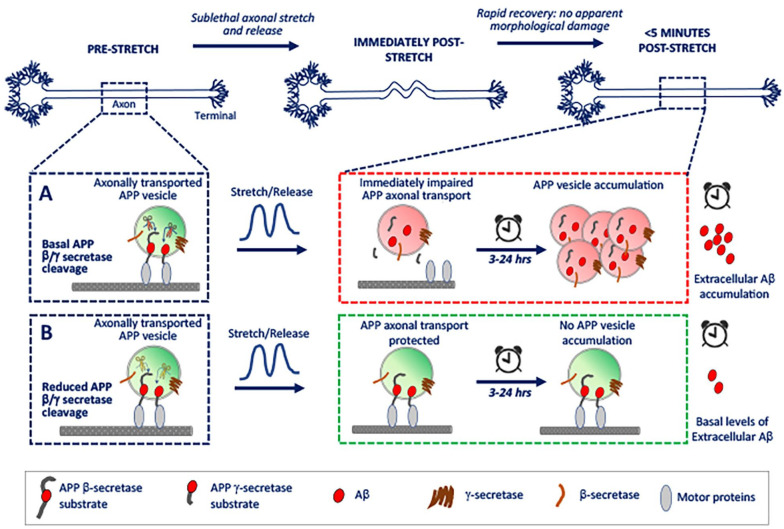
Schematic of early cellular events following mild neuronal deformation. (A) Working model of neuronal response to mild deformation under basal APP cleavage. (B) Working model of neuronal response to mild deformation after reduced APP cleavage, imposed genetically or pharmacologically.

Toward overcoming these challenges, we recently deployed a novel biomechanical device that allowed us to quantitatively assess and visualize APP metabolites formation and axonal transport with high sensitivity in neurons derived from human induced pluripotent stem cells (hiPSCs) that were subject to a mild deformation (ie, stretch). This device recapitulated deformation levels experienced by neurons during concussions.^6^ Our findings provided the first clues for understanding how mild axonal deformation may sow the seeds for future AD-related outcomes. Intriguingly, amyloidogenic cleavage of axonal APP was immediately triggered after sub-lethal deformation, despite maintenance of cell viability, axonal cytoskeletal integrity, and appreciable increases in tau phosphorylation—that is, in the absence of indicators of injury typically associated with more severe traumatic neuronal injury. This places amyloidogenic cleavage of APP as an early and sensitive molecular response to mild mechanical deformation of axons in human neurons. Compellingly, this cleavage in APP instantly and unequivocally inhibited transport of APP along the axon; this then led to aberrant axonal APP accumulations that emerged within hours and were still present for at least 24 hours. Furthermore, levels of secreted Aβ were significantly elevated 24 hours after injury compared to controls, indicating persistently increased amyloidogenic cleavage after injury. These findings, summarized in Figure 1, are consistent with previously established views on APP metabolism and transport, and shape our thinking on pathways of APP-dependent neurodegeneration after traumatic injury in several ways.

First, APP accumulations induced by mild deformation are concomitant with increased Aβ generation.^6^ This in and of itself is not surprising, since as noted above, APP accumulations are a feature of both TBI and AD neuropathology and have long been hypothesized as a potential source for enhanced amyloidogenic processing. The new insight is that such accumulations can occur in the absence of axonal swellings or substantial cytoskeletal disruption within axons, suggesting they are not simply a byproduct of structural damage, but a reliable and sensitive indicator for mild axonal injury. Second, though suspected based on accumulation formation, axonal transport has not been directly visualized following mechanically induced injury. Observed cessation of APP axonal transport in real time places APP axonal transport as one of the earliest observed downstream responses to neuronal injury.^6^ Transport defects were not generalized; transport of other non-APP-dependent vesicular microtubule-based cargoes (eg, lysosomes) were unperturbed, supporting the observation that cytoskeletal tracks were largely intact after mild deformation of axons, and that APP-dependent axonal transport is uniquely sensitive to injury. Third, the mitigation of APP transport defects by inhibition of amyloidogenic processing of APP prior to injury is noteworthy. This protection was validated both pharmacologically with β- and γ-secretase inhibitors, as well as genetically in neurons expressing a CRISPR-engineered mutation that renders APP resistant to β-secretase mediated cleavage.^6^ A key conclusion of this finding is that APP processing and APP axonal transport pathways are interlinked bi-directionally following mild injury. Deformation-induced amyloidogenic APP processing precludes axonal transport of APP-containing vesicles (and thus also accumulation formation),^6^ presumably by severing APP C-terminal kinesin connectivity.^7^ Impaired transport of APP-positive vesicles may in turn feed forward to enhance amyloidogenic cleavage of APP at sites of accumulation, via co-transported β- and γ-secretases in these cargoes.^7,8^

The above concepts were placed in a more “human-relevant” context by the deployment of a naturally occurring genetic variant of *APP, APP*^Icelandic^, which has been posited to protect against AD by reducing β-cleavage of APP.^9^ In particular, mild deformation of hiPSC-neurons expressing this variant was found to attenuate injury-induced transport defects and amyloidogenesis.^6^ This finding suggests that not only is *APP*^Icelandic^ able to reduce aging-associated AD, but it may also protect against AD-related outcomes enhanced by environmental factors—that is, other varieties of sporadically emerging late onset AD, including mild injury. More generally, this finding sets the stage to explore the contribution of individual genomes to the development of AD-related phenotypes after TBI.

## Implications for Future Mechanistic Studies, Diagnosis, and Treatment

The links between mild injury, Aβ processing, and axonal transport warrant deeper mechanistic examination, as they could provide new and potentially transformative targets for diagnosing or modulating injury response pathways. From a biophysical perspective, understanding how exactly a modest, non-lethal mechanical signal acutely and robustly induces secretase activity (ie, how does stretch induce APP cleavage?) is a fascinating question, with additional implications for understanding the fundamental mechanobiology of enzymatic processing. Though much remains to be clarified about the regulation of APP cleavage by the membrane-associated β- and γ-secretases, it is compelling that lipid rafts and/or cholesterol—which affect the mechanical stiffness of the plasma membrane—affect the localization and activity of both secretases.^10,11^ Given that these membrane characteristics are hypothesized to affect the proximity of APP and its secretases as well as the conformational flexibility of the proteolytic enzymes, it is not unreasonable to posit that a stretch-associated mechanical signal may analogously modulate enzymatic activity.

The subcellular localization of APP processing induced by injury is another avenue for further investigation. In our study, Aβ production and processing machinery were indeed observed inside the axon after deformation; however, APP processing triggered by mild injury remains to be examined in the cell body and dendrites, which also might experience mechanical-induced deformation. The interconnectivity among these compartments with respect to APP metabolites is complex, but also essential for understanding upstream modulators and downstream effectors of amyloidogenic pathways. Regulation of cargo transit from the cell body into an axon (or dendrite) has a direct impact on the number of APP containing vesicles entering a given neurite. Within this supply, the identity of a given cargo (eg, an APP-positive vesicles with or without its secretases) and thus also its potential for producing Aβ is defined by the routing of APP and/or its processing machinery through the secretory pathway, their transport to the cell membrane, and their subsequent endocytosis into neurite-bound cargoes.^12^ Further, given lines of evidence demonstrating that endocytosis may be modulated by changes in plasma membrane tension (such as those created by mechanical loading),^13^ an intriguing question is to what extent mild injury impacts the identity of transported APP vesicles through modifying endocytic pathways and if so, the extent of any impact beyond that resulting from APP processing and transport impairments.

Finally, two-dimensional (2D) cultures derived from hiPSC capably enabled the evaluation of human neuron-specific responses to injury using highly sensitive imaging and biochemical approaches. However, it will be important to probe key questions and outcomes in more complex model systems, toward more confident translational relevance. In vitro systems could be extended to include additional cell types impacted by mTBI, including astrocytes and microglial cells. Such cells may be readily integrated with neurons in 2D co-cultures, to interrogate paracrine regulation of AD-associated pathways affected by mild deformation. However, such models do not provide the three-dimensional (3D) connectivity of the central nervous system and cannot be maintained for long time in culture. Recently, tremendous advances have been made in the generation and long-term viability of hiPSC-derived human cerebral organoids, self-organizing 3D structures that recapitulate features of the complex multicellular architecture of the human brain.^14^ These features allow for the examination of longer-term biological and physiological outcomes that are hardly attainable in 2D culture systems, including oligomerization or aggregation of Aβ, tau pathology, or secondary consequences of microglial activation and inflammatory stress on cellular function. Beyond providing mechanistic insight, there is imminent translational value of human brain organoids as high-throughput constructs for diagnostic or therapeutic evaluation,^15^ including in the context of repeated or more severe injury. Despite their advantages, though, organoids cannot fully reproduce the systemic complexity of the in vivo response to mTBI. Thus, outcomes in organoids must ultimately be integrated with outcomes from animal models of mTBI and data from patients, including in vivo animal models, biomarkers collected from human subjects, and clinical outcomes. Such a combinatorial approach would provide a fuller picture of both human-specific and whole-organism level impacts of concussion or mTBI-associated outcomes.

Collectively, the above data suggest that APP amyloidogenic processing may act as a sensitive “neuronal damage biomarker.” From a diagnostic perspective, developing nano-sensors to monitor such processing may provide a surrogate for tracking the cellular consequences of mild axonal deformation. In addition, the linkage between amyloidogenic pathways and axonal transport suggests that intervening in APP amyloidogenic processing pathways prior to injury (eg, before engaging in a contact sports) or immediately after an injury event, may be a viable strategy for therapeutic protection against stretch-induced neuronal dysfunction. These findings open additional lines of investigation for future translational work.
